# The calcium dynamics of human dental pulp stem cells stimulated with tricalcium silicate-based cements determine their differentiation and mineralization outcome

**DOI:** 10.1038/s41598-020-80096-5

**Published:** 2021-01-12

**Authors:** Elanagai Rathinam, Srinath Govindarajan, Sivaprakash Rajasekharan, Heidi Declercq, Dirk Elewaut, Peter De Coster, Luc Martens, Luc Leybaert

**Affiliations:** 1 Department of Paediatric Dentistry and Special Care, PAECOMEDIS Research Cluster, Ghent University, Ghent University Hospital, 9000 Ghent, Belgium; 2 Department of Internal Medicine and Paediatrics, Ghent University, Ghent University Hospital, 9000 Ghent, Belgium; 3 Unit for Molecular Immunology and Inflammation, VIB-Center for Inflammation Research, Technologiepark 71, 9052 Zwijnaarde, Ghent, Belgium; 4 Tissue Engineering and Biomaterials Group, Department of Human Structure and Repair, Ghent University, Ghent University Hospital, 9000 Ghent, Belgium; 5 Tissue Engineering Lab, Department of Development and Regeneration, KU Leuven, 8500 Kortrijk, Belgium; 6 Department of Reconstructive Dentistry and Oral Biology, Dental School, Ghent University, Ghent University Hospital, 9000 Ghent, Belgium; 7 Department of Basic And Applied Medical Sciences - Physiology Group, Ghent University, Ghent, Belgium

**Keywords:** Calcium signalling, Biomaterials - cells, Mesenchymal stem cells

## Abstract

Calcium (Ca^2+^) signalling plays an indispensable role in dental pulp and dentin regeneration, but the Ca^2+^ responses of human dental pulp stem cells (hDPSCs) stimulated with tricalcium silicate-based (TCS-based) dental biomaterials remains largely unexplored. The objective of the present study was to identify and correlate extracellular Ca^2+^ concentration, intracellular Ca^2+^ dynamics, pH, cytotoxicity, gene expression and mineralization ability of human dental pulp stem cells (hDPSCs) stimulated with two different TCS-based biomaterials: Biodentine and ProRoot white MTA. The hDPSCs were exposed to the biomaterials, brought in contact with the overlaying medium, with subsequent measurements of extracellular Ca^2+^ and pH, and intracellular Ca^2+^ changes. Messenger RNA expression (BGLAP, TGF-β, MMP1 and BMP2), cytotoxicity (MTT and TUNEL) and mineralization potential (Alizarin red and Von Kossa staining) were then evaluated. Biodentine released significantly more Ca^2+^ in the α-MEM medium than ProRoot WMTA but this had no cytotoxic impact on hDPSCs. The larger Biodentine-linked Ca^2+^ release resulted in altered intracellular Ca^2+^ dynamics, which attained a higher maximum amplitude, faster rise time and increased area under the curve of the Ca^2+^ changes compared to ProRoot WMTA. Experiments with intracellular Ca^2+^ chelation, demonstrated that the biomaterial-triggered Ca^2+^ dynamics affected stem cell-related gene expression, cellular differentiation and mineralization potential. In conclusion, biomaterial-specific Ca^2+^ dynamics in hDPSCs determine differentiation and mineralization outcomes, with increased Ca^2+^ dynamics enhancing mineralization.

## Introduction

Tricalcium silicate-based (TCS-based) cements are hydraulic bioactive materials widely used as endodontic cements in dentistry and as bone substitutes in orthopedics^1^. Several commercial TCS-based cements with subtle modifications in the manufacturing process and composition are available. ProRoot White MTA (WMTA) (Dentsply, Tulsa Dental, OK, USA) and Biodentine (Septodont, Saint-Maur-des-Fossés, France) are two representative TCS-based cements with superior clinical success in dentistry. The widespread clinical indications of TCS-based cements are primarily based on their ability to form calcium hydroxide as a by-product of hydration^2^. The subsequent dissolution of calcium hydroxide to release hydroxide (OH^-^)and calcium ions (Ca^2+^) creates a desirable environment to promote healing and repair of soft and hard tissues^3,4^. While hydroxide ions create an alkaline environment responsible for antibacterial and anti-inflammatory activity, Ca^2+^ ions play a comprehensive role as a vital intracellular second messenger that governs diverse cellular processes such as gene transcription, protein expression, cell proliferation, differentiation, apoptosis, and activation of excitatory cell types^5,6^.

TCS-based cements release Ca^2+^ in the microenvironment causing elevated extracellular Ca^2+^ concentration and as a consequence transiently increase the intracellular Ca^2+^ concentration due to calcium-sensing receptor (CaSR) activation^7,8^. The dynamic Ca^2+^ dependent signalling system can affect numerous Ca^2+^ sensitive enzymes that convert changes in extracellular Ca^2+^ concentration and intracellular Ca^2+^ dynamics into well-defined cell actions^9^. Analysis of the molecular cues embedded in the intracellular Ca^2+^ dynamics will lead to better understanding of the intracellular signalling mechanisms involved in regulating the bioactivities of cells^10^. Intracellular Ca^2+^ dynamics play a dual role by acting both as an initiator and mediator of stem cell differentiation. Thorough knowledge on the role of Ca^2+^ dynamics in the differentiation of stem cells into a tissue-specific lineage may offer an alternative biotechnological approach to exploit the unique properties of stem cells^10^.

Calcium signalling is multifaceted and depends on the cell type^9^. Although Ca^2+^ signalling plays an indispensable role in dental pulp and dentin regeneration, there is a lack of information on the Ca^2+^ dynamics of human dental pulp stem cells (hDPSCs). The aim of the present study was to identify and correlate extracellular Ca^2+^ concentration, intracellular Ca^2+^ dynamics, pH, cytotoxicity, gene expression and mineralization ability of human dental pulp stem cells (hDPSCs) stimulated with two different TCS-based biomaterials; ProRoot WMTA and Biodentine. Our work shows significant differences in extracellular and intracellular Ca^2+^ changes that link to distinct patterns of hDPSCs gene expression, cellular differentiation and mineralization potential.

## Results

### Calcium release and pH

**Figure 1:**
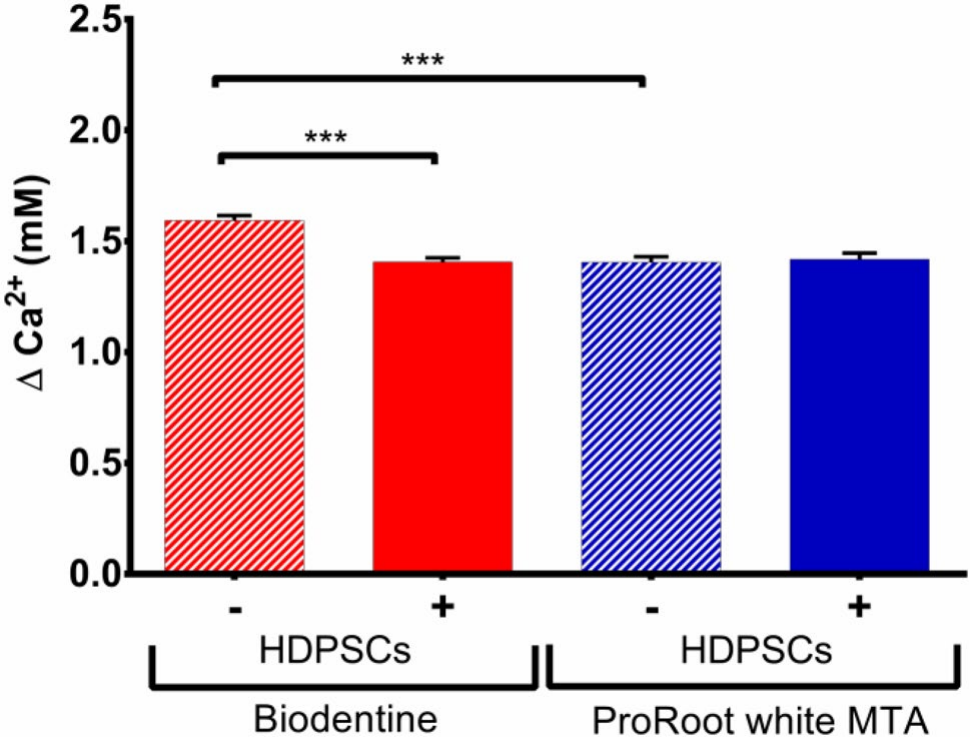
Changes in extracellular Ca^2+^ ion concentration (mM) in α—MEM with and without hDPSCs after 30 min biomaterial exposure. One way ANOVA with Tukey post-hoc comparisons showed significantly higher Ca^2+^ released by Biodentine than ProRoot WMTA (*p* < 0.001) when no cells were present. In the presence of hDPSCs, the larger Biodentine-triggered extracellular Ca^2+^ increase was significantly reduced to the level observed with ProRoot WMTA (*p* < 0.001).

Ca^2+^ measurements in the cell medium, in response to exposure to TCS-based cements in the absence as well as presence of hDPSCs were performed (Fig. 8). In the absence of cells, Biodentine released significantly more Ca^2+^ in the α-MEM medium than ProRoot WMTA (*p* < 0.001) (Fig. 1); no significant difference in pH was found between the groups (Supplementary Fig. 1). Interestingly, in the presence of hDPSCs in the culture dishes, the larger Biodentine-linked Ca^2+^ release in the medium was reduced to the level observed with ProRoot WMTA (*p* < 0.001), indicating that the cells take up the extra Ca^2+^ load provided by Biodentine (Fig. 1).

### Intracellular calcium

**Figure 2:**
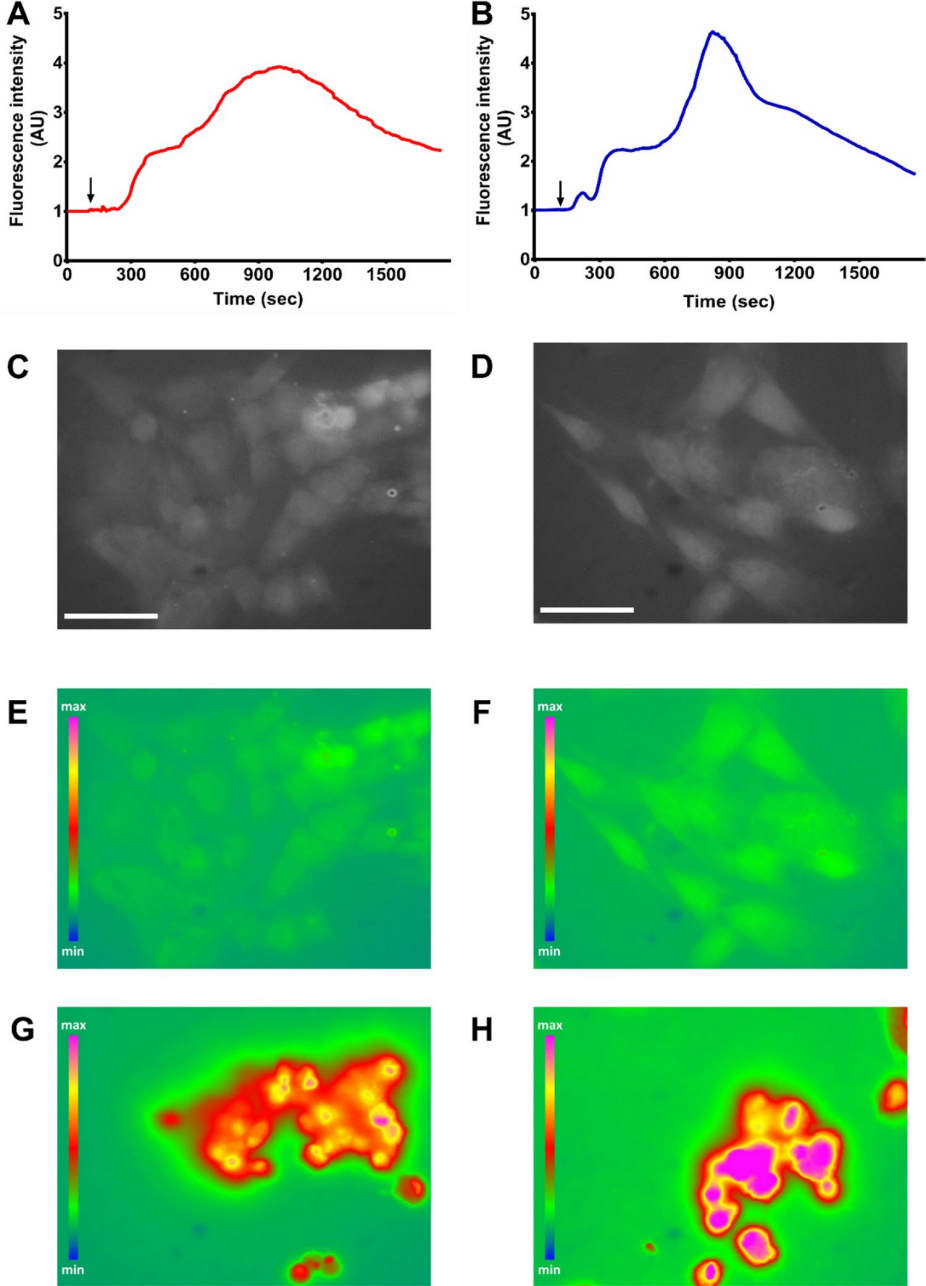
Live cell Ca^2+^ imaging in hDPSCs. (**A**, **B**) show representative time courses of gross intracellular Ca^2+^ dynamics for Biodentine (**A**) and ProRoot WMTA (**B**) induced by the two biomaterials (averaged signal over all cells in view). Arrows indicate exposure to the biomaterial lowered to contact the recording medium. (**C**,**E**,**G**) show representative Ca^2+^ images for Biodentine, with (**C**) representing a B/W image of stem cells loaded with fluorescent Ca^2+^ indicator to reveal the cells, (**E**) a pseudocolored Ca^2+^ image taken before biomaterial exposure and (**G**) a Ca^2+^ image at the peak of the global Ca^2+^ change. (**D**,**F**,**H**) show corresponding images for exposure to ProRoot WMTA. Scale bar measures 50 µm; pseudocolor scale indicates fluorescent Ca^2+^ indicator signal level.

Ca^2+^ imaging experiments to determine the cellular responses to biomaterial exposure and its associated changes in extracellular Ca^2+^ were performed. Cells with acetylcholine (1 µM) were challenged to verify their responsiveness. Oscillatory changes in intracellular Ca^2+^ that are typical for hDPSCs were found (Supplementary Fig. 2). Subsequently, cells were exposed to TCS-based cements placed in an insert, which after baseline Ca^2+^ recording, was lowered to contact the bathing solution under continuous Ca^2+^ imaging (Fig. 2). Gross analysis of the intracellular Ca^2+^ signal averaged over all cells in view, demonstrated a Ca^2+^ increase characterized by a peak followed by recovery (Fig. 2).

**Figure 3:**
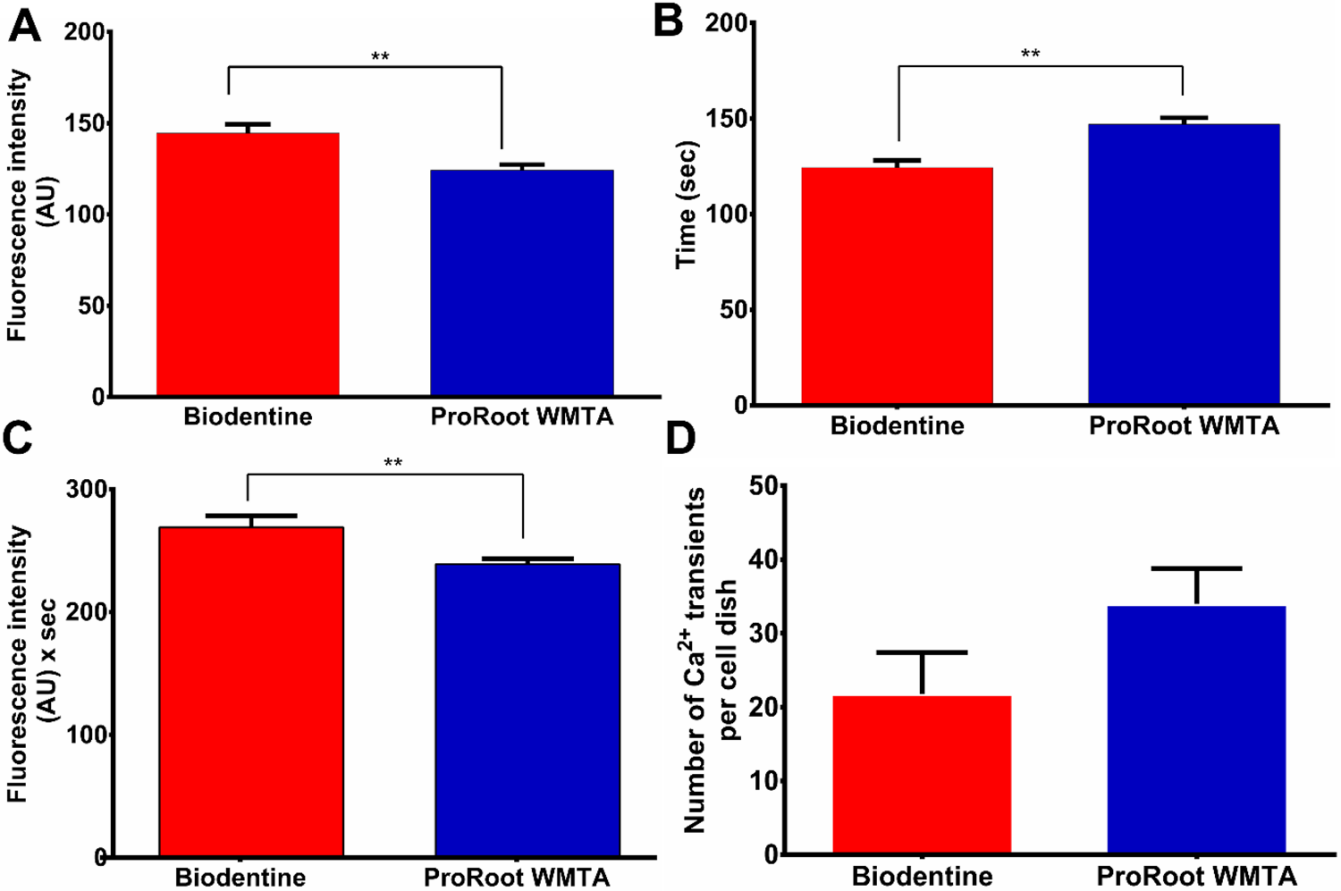
Properties of cellular Ca^2+^ dynamics in response to biomaterial exposure. (**A**) Peak amplitude of Ca^2+^ changes. Unpaired t test with Welch's correction shows significantly higher peak amplitude in the Biodentine group (*p* < 0.0001). (**B**) Time to maximum amplitude of the Ca^2+^ change. Unpaired t test with Welch's correction shows significantly increased time to max amplitude in the ProRoot WMTA group (*p* < 0.0001). (**C**) Area under the curve of the Ca^2+^ changes. Unpaired t test with Welch's correction shows significantly higher area under the curve in the Biodentine group (*p* < 0.01). (**D**) Number of Ca^2+^ transients per cell dish.

Finer grained analysis of the Ca^2+^ dynamics in individual cells, in response to TCS challenging with the two biomaterials were performed. This showed **that Biodentine produced a significantly higher maximum amplitude of the Ca^2+^ transients (*p* < 0.0001) (Fig. 3A), a reduced time to reach the maximum (*p* < 0.0001) (Fig. 3B) and an increased area under the curve (*p* < 0.01) (Fig. 3C) as compared to ProRoot WMTA. The number of cellular Ca^2+^ transients per cell dish did not differ significantly between the groups (Fig. 3D). On average, the number of Ca^2+^ transients per cell after biomaterial challenging was low, amounting to 0.92 transients per 10 min for Biodentine and 1.12 for ProRoot WMTA (~ 3 transients over 30 min for both).

Control experiments under conditions of cell pre-loading with the intracellular Ca^2+^ chelator BAPTA-AM were performed. Complete absence of intracellular Ca^2+^ dynamics upon exposure to the two biomaterials were found (Supplementary Fig. 3).

### Gene expression

**Figure 4:**
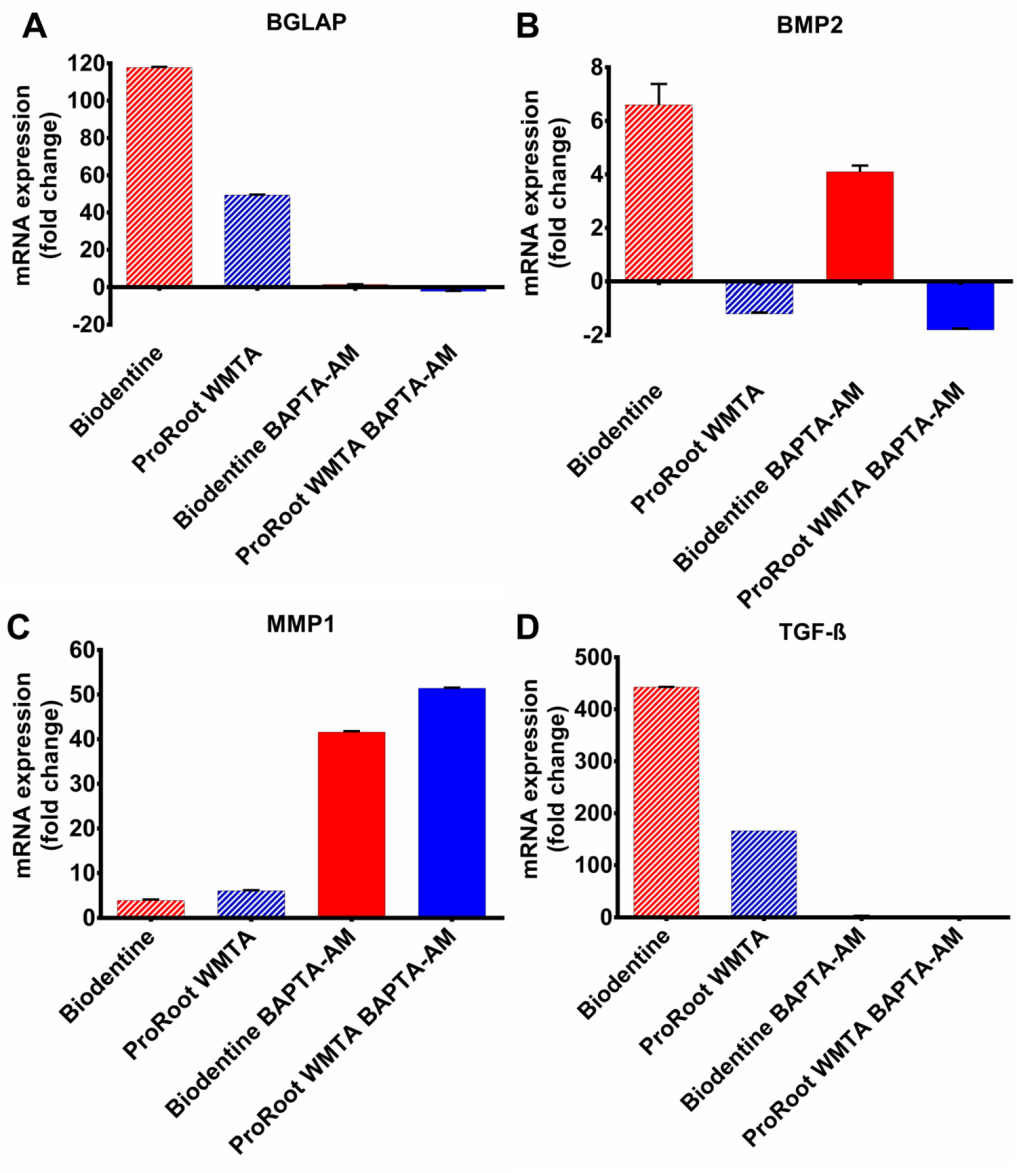
qRT-PCR expression. mRNA expression of (**A**) BGLAP, (**B**) TGF-β, (**C**) MMP1 and (**D**) BMP2 1 day after biomaterial exposure. Histogram shows up-regulated and down-regulated mRNA expression in relative fold change. Details of specific primers used for gene expression analysis are provided in Table 1.

Elevations in extracellular Ca^2+^ concentrations^11^ as well as intracellular Ca^2+^ changes are well known to influence gene expression^12^. The response of four specific gene markers were tested: BGLAP, TGF-β, MMP1 and BMP2 to the two TCS-based biomaterials. Biodentine exposure for 1 day resulted in upregulation of BGLAP and TGF-β that was more than twice as large compared to ProRoot WMTA (Fig. 4A,B); only small differences were observed for MMP-1 (Fig. 4C). BMP2 was upregulated by Biodentine but ProRoot WMTA downregulated the gene (Fig. 4D), demonstrating again a pronounced difference between the two TCS-based cements. These experiments were repeated under conditions of intracellular Ca^2+^ chelation by loading the cells with 1 µM BAPTA-AM which also present during the 1 day exposure to the TCS-based cements. Such treatment clearly suppressed the BGLAP and TGF-β responses observed with both biomaterials. By contrast, the small changes observed for MMP1 became more pronounced in BAPTA-AM treated cells. For BMP2, the effects were intermediate, with a reduction by one third of the Biodentine induced upregulation and a one third increased downregulation in response to ProRoot WMTA (Fig. 4D).

### Experiments on cell death

**Figure 5:**
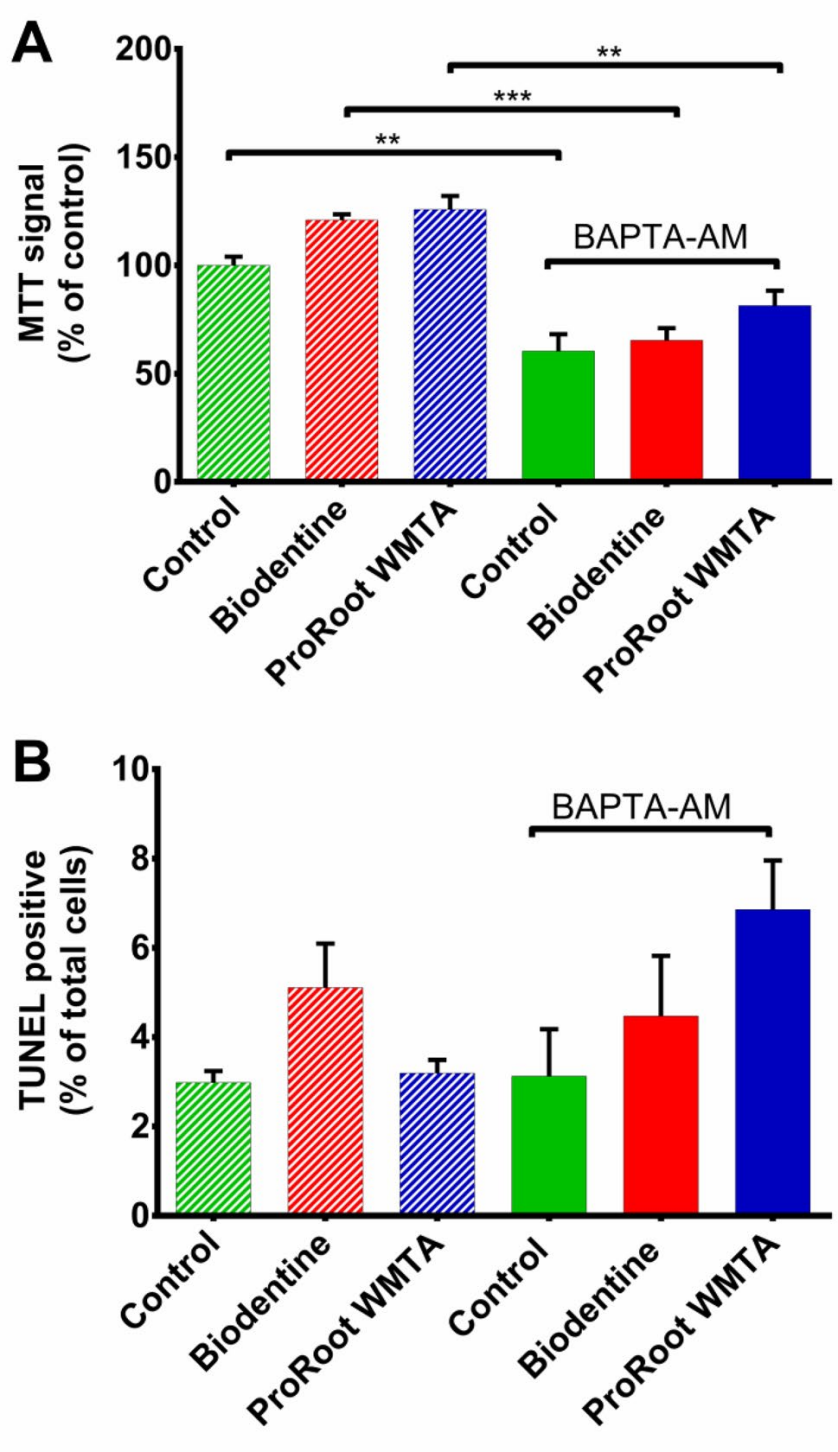
Cytotoxicity assay of Human Dental Pulp Stem Cells (hDPSCs). (**A**) MTT assay after 1 day exposure to the biomaterials. One way ANOVA with Tukey post-hoc comparisons showed that intracellular buffering by addition of BAPTA-AM significantly reduced the MTT signal in Control (*p* < 0.01), Biodentine (*p* < 0.001) and ProRoot WMTA (*p* < 0.01) groups. (**B**) TUNEL assay after 1 day exposure to the biomaterials. One way ANOVA with Tukey post-hoc comparisons revealed no significant difference in the % of TUNEL positive cells.

Subsequently, it was verified whether cell survival/cell death was affected by any of the treatments used in the experiments on gene expression. Viability as measured with the MTT assay demonstrated that 1 day exposure to the TCS-based cements did not result in significant differences compared to control (Fig. 5A). However, BAPTA-AM treatment (as applied in the gene studies) significantly decreased the MTT signal (*p* < 0.01) (Fig. 5A). Further investigations with the TUNEL assay (Fig. 5B) and live/dead staining (Supplementary Fig. 4) did not reveal any difference in terms of cell death, suggesting that the BAPTA-AM induced decrease in MTT signal is most likely caused by suppression of mitochondrial metabolic activity resulting from the dampening effect of BAPTA-AM on intracellular Ca^2+^ dynamics and not resulting from cell death^13^.

### Mineralization assay

**Figure 6:**
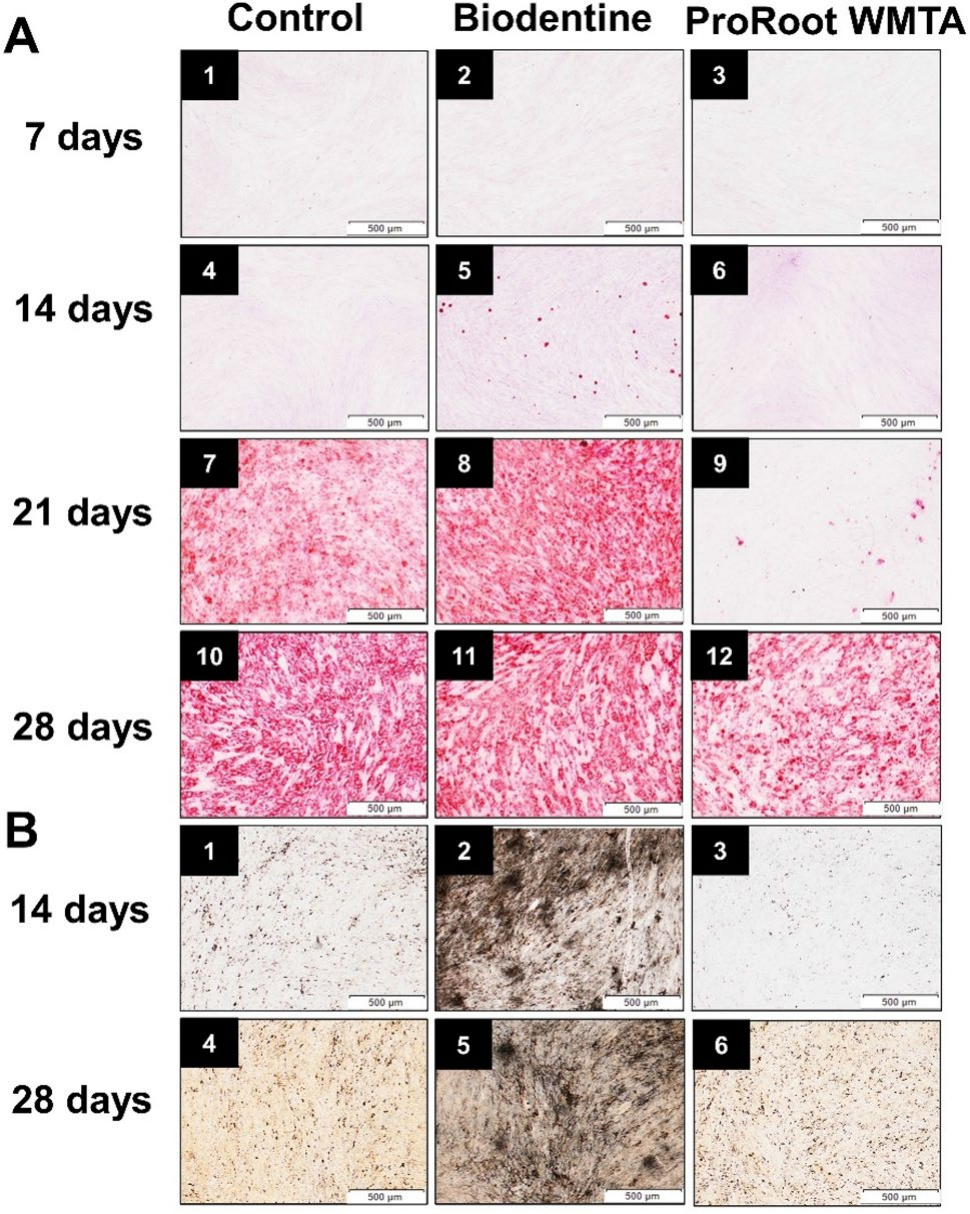
Mineralization assay to detect Ca^2+^ deposits. Alizarin red staining of specimens after 7 (A1-3), 14 (A4-6), 21 (A7-9) and 28 (A10-12) days. Von Kossa staining of specimens after 14 days (B1-3) and 28 days (B4-6). Column 1, 2 and 3 are representative images of Control, Biodentine and ProRoot WMTA respectively. Presence of mineralized nodules in the Biodentine group could be seen starting from 14 days while no mineralization is seen in the ProRoot WMTA group at the same time period. At 21 days, Biodentine shows extensive mineralization while ProRoot WMTA shows scattered presence of mineral nodules comparable to that seen in the Biodentine group at 14 days. Similar results were observed with Von Kossa staining experiments.

Further, the mineralization potential of TCS-based cements was verified. Therefore, hDPSCs cultured in the presence of TCS-based biomaterials were evaluated for 7, 14, 21 and 28 days. Our results show that Biodentine induced faster mineralization (14 days) compared to ProRoot WMTA (21 days). At 21 days, Biodentine showed extensive mineralization while ProRoot WMTA displayed scattered presence of mineral nodules comparable to that seen in the Biodentine group at 14 days (Fig. 6).

## Discussion

TCS-based cements are primarily used in dentistry and their interaction with hDPSCs are of great interest^14^. Potential use of hDPSCs in tissue regeneration by virtue of their ability to differentiate into fibroblasts, osteoblasts, odontoblasts, adipocytes, neurogenic and myogenic tissues in vitro make them a favorable model system for studying Ca^2+^ signals^15^. hDPSC derived odontoblasts play an active role in the transport and accumulation of Ca^2+^ which leads to regeneration of the dentin-pulp complex^16^. In this study, custom prepared hDPSCs with 96% purity, extracted by an enzyme digestion method from healthy unerupted human third molars were used. Transwell membranes with a pore diameter of 0.4 µm were used to mimic the absence of direct contact between the biomaterial and hDPSCs while allowing for soluble compounds from the biomaterials to reach the cells, similar to clinical conditions^17^.

TCS-based cements are known to release Ca^2+^ in varying concentration, depending on the cement composition. In this study, Biodentine released significantly more Ca^2+^ than ProRoot WMTA after 30 min in α-MEM medium (*p* < 0.001). This could be explained by the fact that despite both ProRoot WMTA and Biodentine contain TCS as their main ingredient, the structure and composition of the two TCS-based cements reveal certain differences that could contribute either directly or indirectly to the difference in Ca^2+^ release. Compared to ProRoot WMTA, Biodentine has a smaller particle size, which is known to increase the Ca^2+^ release^18^. In addition to TCS as the major ingredient, ProRoot WMTA contains dicalcium silicate and tricalcium aluminate^19^. These additional ingredients form minimal or no calcium hydroxide upon hydration leading to a lesser degree of Ca^2+^ release. The fact that the Ca^2+^ release was not accompanied by a corresponding rise in pH suggests that the source of this Ca^2+^ is not only from the formation and dissolution of calcium hydroxide but possibly also from other calcium compounds being present. Biodentine uses calcium chloride as the liquid medium for hydration while ProRoot WMTA uses water. The use of calcium chloride could enhance the Ca^2+^ release by accelerating the setting reaction, increasing calcium hydroxide formation and by Ca^2+^ release from unreacted calcium chloride^20^.

The larger Ca^2+^ release from Biodentine was not detectable in the presence of hDPSCs, indicating that Ca^2+^ is taken-up by the cells. Cellular Ca^2+^ uptake involves subsequent Ca^2+^ binding to proteins and uptake into organelles like the endoplasmic reticulum (ER) and mitochondria^9,21^. This organelle-based Ca^2+^ load may affect gene expression and viability/cell death. In fact, the ER-mitochondrial Ca^2+^ axis is a major player and determinant of cell survival/cell death^22^ and is also known to affect gene expression^23^.

Our results obtained from intracellular Ca^2+^ measurements closely reflect the differences observed between the two TCS-based cements on extracellular Ca^2+^. The significantly larger Ca^2+^ release from Biodentine correlates well with the significantly larger area under the curve of intracellular Ca^2+^ changes (p < 0.01), the significantly higher maximum amplitude (*p* < 0.0001) and the significantly shorter time to maximum change (*p* < 0.0001) observed in Biodentine as compared to ProRoot WMTA (see Fig. 3). The larger Ca^2+^ load associated with Biodentine may act through increased Ca^2+^ entry into the cell, possibly via Cav1.2 L-type Ca^2+^ channels^24,25^, ORAI1, an essential pore subunit of store-operated Ca^2+^ entry (SOCE) channels in stem cells^26^ or TRPM4 channels^27^. Increased Ca^2+^ entry is followed by intracellular cycling between the cytoplasm, ER Ca^2+^ stores and mitochondria in mesenchymal stem cells, thus leading to the observed Ca^2+^ dynamics^28,29^.

Calcium dynamics can activate signalling pathways in both the nucleus and cytoplasm to induce gene expression by different pathways, that can function both as an inhibitor or activator of gene expression^30^. The frequency, duration and amplitude of Ca^2+^ transients are essential for increasing the efficiency and specificity of gene expression^21^. There is a nonlinear relationship between gene transcription and intracellular Ca^2+^ dynamics, that periodically exceeds the threshold for activation of gene transcription^31^. The Ca^2+^ oscillation frequency hereby differentially controls the activation of different genes and direct cells to specific developmental pathways^32^. The present experiments did not reveal prominent oscillatory activity, with only 3 elevations occurring per cell over the 30 min recording period for both biomaterials. This makes it possible that amplitude rather than frequency is most important in the observed effects, as is the case in the slow oscillatory activity leading to oocyte activation^33,34^. In any case, the results revealed that Biodentine promoted mRNA expression of BGLAP, TGF-β, and BMP2 indicating that the increased Ca^2+^ load and intracellular dynamics can enhance the expression of genes associated with odonto-/osteogenic differentiation necessary for successful regeneration of the dentine-pulp complex^35,36^. Intracellular buffering with BAPTA-AM treatment inhibited the expression of BGLAP and TGF-β, thereby supporting an underlying role of Ca^2+^ signalling in the upregulation of these genes. Furthermore, addition of BAPTA-AM reduced the upregulation of BMP2 but resulted in overexpression of MMP1. It is well known that an increase in extracellular Ca^2+^ enhances the expression of BMP2 by activation of the Ca^2+^ sensing receptor, elevation of intracellular Ca^2+^ and stimulation of Ca^2+^/calmodulin-dependent nuclear factor of activated T cells (NFAT) signalling pathways^7^.

Calcium plays a significant role in molecular processes responsible for mediating cell survival and death, including defence and programmed cell death mechanisms, such as cell cycle, apoptosis, and autophagy^37,38^. In the present study, combined MTT and TUNEL assays revealed absence of cytotoxicity after 1 day exposure to the biomaterials. Moreover, BAPTA-AM treatment caused suppression of mitochondrial metabolic activity causing a decrease in the MTT signal in all the groups. Inconsistent results regarding cell death were seen in the literature where Biodentine performed better^39^, similar^40–43^ or worse^44^ than MTA. Such contradictory results may be related to the type of target cells, method of cytotoxicity assessment, direct contact of cells with the materials and concentration of materials. Three-dimensional culture of dental pulp stem cells in direct contact to Biodentine and MTA revealed higher cell viability compared to ProRoot MTA after 1, 3 5 and 7 days^45^. In the study by Daltoe et al., serial dilutions (1:1, 1:10 and 1.100) of extracts from Biodentine and ProRoot MTA showed no significant differences in cell viability after 1 and 2 days in any of the concentrations tested^46^. The cytotoxic effect of Biodentine and ProRoot MTA on hDPSCs were concentration dependent as cell viability was higher at 1:100 concentration compared to 1:10 or 1:1 concentration. Similar results were obtained when hDPSCs were cultured in medium conditioned with Biodentine or ProRoot MTA^47^. When transwell membrane was used to avoid direct contact between the biomaterials and hDPSCs, there was no significant difference in cell viability and cell migration between ProRoot MTA and Biodentine^48^.

Further, the effects of TCS-based cements on the mineralization potential of hDPSCs were investigated using the Alizarin red and Von Kossa staining technique. Biodentine induced faster and increased mineralized nodule formation compared with the other groups, consistent with previous literature^42,49,50^. The increased mineralization potential of Biodentine is in conformity with its larger material-linked Ca^2+^ discharge, the larger intracellular Ca^2+^ response and the upregulation of BMP2, BGLAP and TGF-β observed in our study. As Ca^2+^ is deposited by osteoblasts and/or odontoblasts, the present results also highlight the osteogenic and/or odontogenic differentiation potential of hDPSCs in the presence of Biodentine and ProRoot WMTA. In agreement with the findings of the present study, previous literature suggests that in comparison to ProRoot MTA, Biodentine demonstrated significantly better cell survival and proliferation of osteoblasts and periodontal ligament cells^39,51^.

Limitations to simulate the complex biological conditions of the clinical situation exist and hence the results obtained from the present study must be observed with caution for direct correlation with clinical scenarios. Calcium sensitive dyes are widely used for evaluating Ca^2+^ signalling, but their applications have certain drawbacks. Special conditions are required for loading of the cells, bleaching may occur because of extended imaging periods and intracellular dye accumulation may increase cytoplasmic Ca^2+^ buffering^52^.

It is essential to expand our knowledge on the numerous pathways by which Ca^2+^ regulates cellular functions^9^. A typical characteristic of Ca^2+^ signalling is the manner by which different Ca^2+^ signals can be translated into specific cell functions depending on the type of signal^6^. The wide range of functions executed by Ca^2+^ is attributed to the versatility in speed, amplitude, duration and spatiotemporal pattern of Ca^2+^ signals as well as by interactions between Ca^2+^ and other signalling pathways most likely mediated by different cellular processes^53^. Further research on the versatile patterns of Ca^2+^ signals is essential for studying the mechanisms lying beyond the cellular functions^5^.

## Conclusion

Our work demonstrates that hDPSCs take up the Ca^2+^ released from TCS-based biomaterials without provoking cell death. The larger Biodentine-linked Ca^2+^ load was reflected in altered intracellular Ca^2+^ dynamics, which consequently resulted in differential gene expression, cellular differentiation and mineralization potential of hDPSCs stimulated with TCS-based cements.

## Materials and methods

### Isolation of stem cells

**Figure 7:**
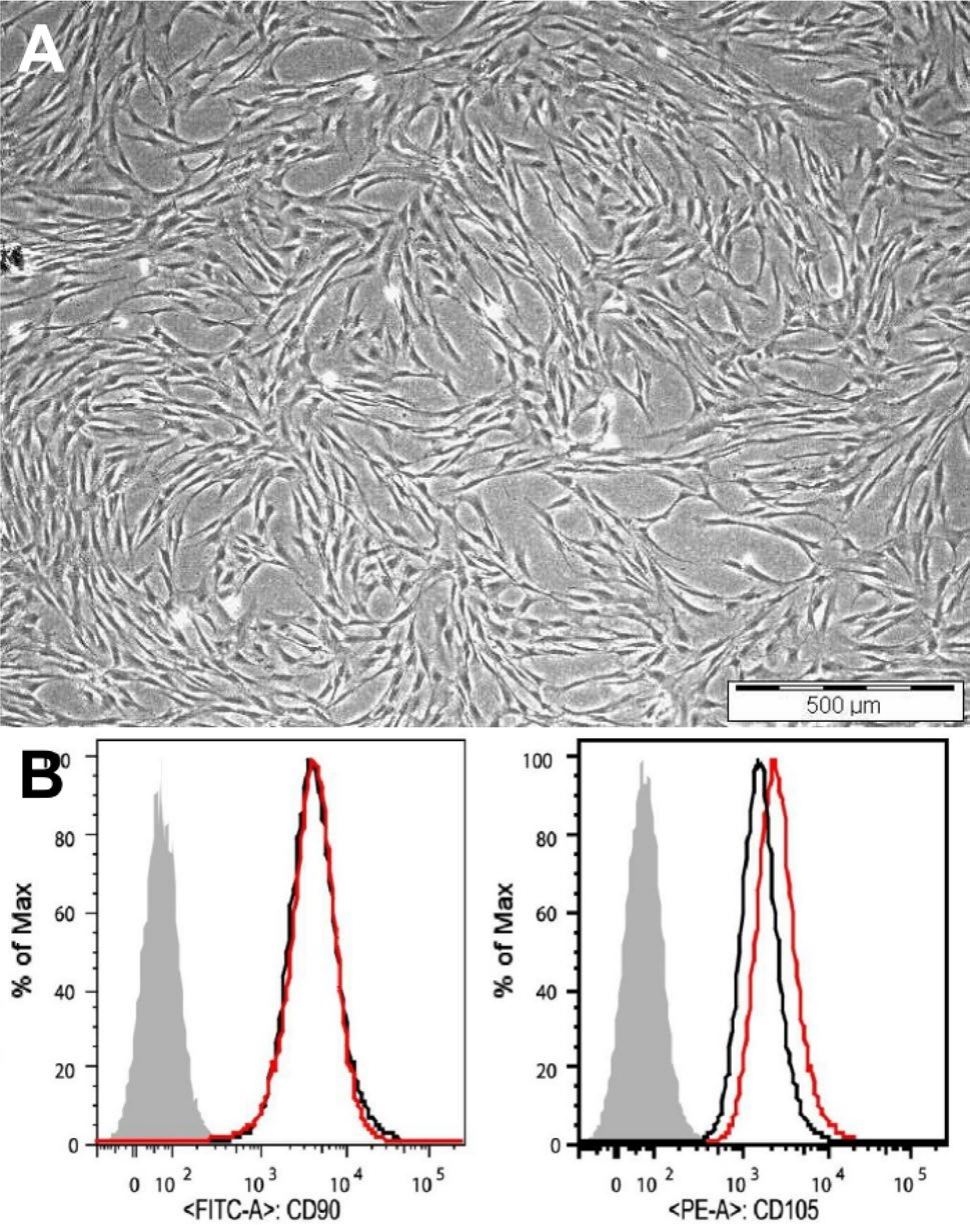
Characterization of isolated Human Dental Pulp Stem Cells (hDPSCs) (**A**) Representative phase contrast microscopic image of hDPSCs. (**B**) Flow cytometry histograms of specific markers (CD90 and CD105) expressed in custom prepared hDPSCs extracted by enzyme digestion method showed a purity of 96%.

Human Dental Pulp Stem Cells (hDPSCs) were extracted from unerupted human third molars by enzyme digestion^54^. Written informed consent was collected from all patients and ethical approval was obtained from the Ethical Committee of University hospital, Ghent, Belgium according to laws of ICH Good Clinical Practice (GE11-LM-go-2006/57). The tooth crown was cleaned with iodine and 70% ethanol. The tooth was then cut with a bone cutter at the cemento-enamel junction to remove the pulp tissue and digested with type 1 collagenase and dispase. Cell suspension was cultured in a 25cm^2^ flask in Alpha modified Eagles medium (α-MEM, Sigma-Aldrich, Overijse, Belgium) with 10% fetal bovine serum and antibiotics (100U/ml Pencillin and 100 mg/ml streptomycin) at 37 °C and 5% CO_2_. Flow cytometry analysis was performed to identify the purity of the stem cell culture obtained. Purity of the custom prepared stem cells were determined by mesenchymal stem cell markers CD90, and CD105. Custom prepared hDPSCs were grown to subconfluence and attained a purity of 96% (Fig. 7).

### Cell culture

For the experiments, hDPSCs were seeded in a 24 well plate at a density of 40,000 cells/well (for Ca^2+^ release, pH, cytotoxicity and mineralization assay), in a 6 well plate at a density of 5 × 10^5^ cells/well (for qRT-PCR), or in 35 mm glass bottom dishes (MatTek Corporation, Massachusetts, USA) at a density of 1 × 10^5^ (for live Ca^2+^ fluorescence imaging). For intracellular buffering 1 µM of BAPTA-AM (Life technologies, California, USA) was added to α-MEM medium. All groups were maintained at 37 °C and 5% CO_2_ for 1 day.

### Sample preparation

**Figure 8:**
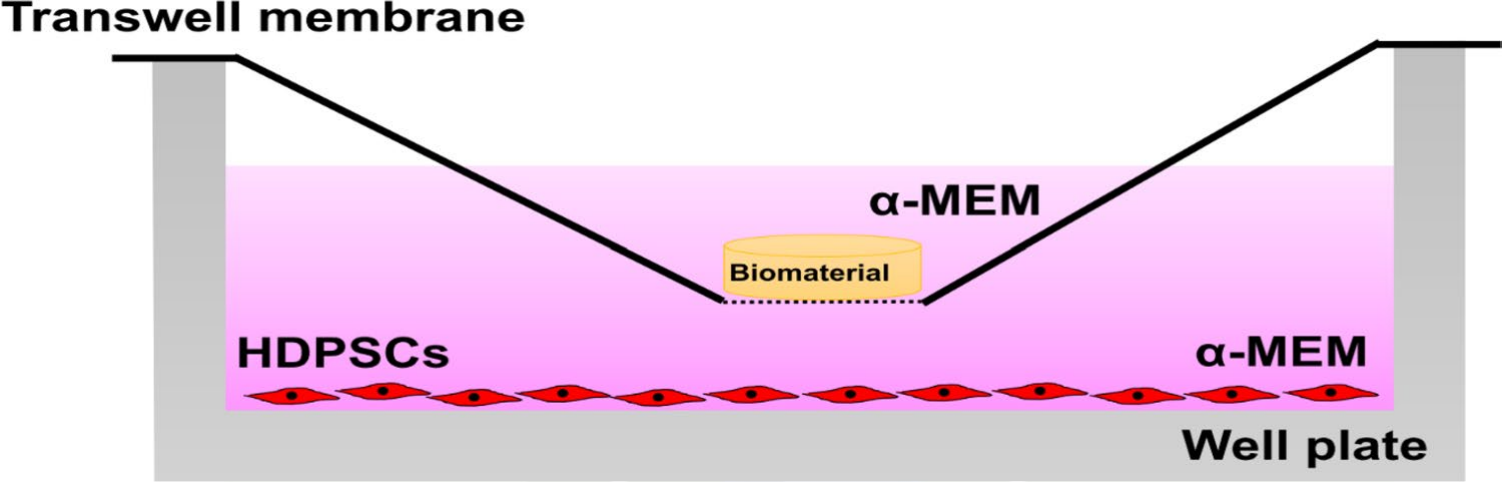
Positioning of biomaterial in the transwell membrane without direct contact with hDPSCs.

Biodentine (Septodont, Saint-Maur-des-Fossés, France) and ProRoot White MTA (WMTA) (Dentsply, Tulsa Dental, OK, USA) were mixed according to manufacturer’s instructions and condensed in teflon moulds of height 1 mm and diameter 5 mm (for Ca^2+^ release, pH, live Ca^2+^ fluorescence imaging and cytotoxicity) or height 2 mm and diameter 8 mm (for qRT-PCR). Although the size of samples was different, the ratio of the exposed surface area of the sample to the volume of the surrounding medium (mm^2^/ml) was maintained as a constant in all experiments. The samples were allowed to set for 3 h in 100% relative humidity at 37 °C and sterilized by ultraviolet radiation for one hour. Samples were placed in transwell inserts of 0.4 µm pore size (Greiner bio-one, Kremsmünster, Austria) to avoid direct contact of the biomaterial with hDPSCs (Fig. 8). Positive control (without biomaterial), Biodentine and ProRoot WMTA groups were evaluated with/without BAPTA-AM loading. For cytotoxicity assay, qRT-PCR and mineralization assay, a sample size of n = 3/group were used while n = 6/group were used for Ca^2+^ release, pH and live Ca^2+^ fluorescence imaging experiments.

### Calcium release and pH

Calcium release was measured by use of a Ca^2+^ ion selective combination electrode (Hanna HI 4004, Hanna Instruments, Temse, Belgium). To reduce interferences and the formation of refractory oxides, 40 µl ionic strength adjuster (1 M KCl ISA buffer, HI 4004–00, Hanna Instruments, Temse, Belgium) was added to 1 ml of all sample solutions. The electrode was calibrated using a standard calibration line. The pH of the solutions was measured with a pH glass electrode (Primatrode, Metrohm, Switzerland). Calcium release from the biomaterial and pH were measured both in the presence and absence of hDPSCs.

### Live cell Ca^2+^ imaging

For live cell Ca^2+^ imaging, hDPSCs were loaded with 5 µM of Fluo-3-AM (Invitrogen, California, USA) and 0.01% pluronic F127 (Invitrogen, California, USA) at room temperature in the dark and incubated for 1 h. The cells were then washed and de-esterified for 15 min in HBSS (Hanks Balanced Salt Solution) – HEPES (4-(2-hydroxyethyl)-1-piperazineethanesulfonic acid) buffer (0.14 g/l cacl_2_.2H_2_O (1.8 mM) containing 0.2 g/l MgSO_4_.7H_2_O (0.81 mM), 0.032 g/l Na_2_HPO_4_.2H_2_O (0.18 mM), 0.4 g/l KCl (5.36 mM), 0.06 g/l KH_2_PO_4_ (0.44 mM), 1.0 g/l D-glucose (5.55 mM), 5.95 g/l HEPES (25 mM) with PH 7.4) with a Ca^2+^ concentration of 1.8 mM similar to α-MEM. Cells were superfused with HBSS-HEPES buffer for 2 min and were then challenged with acetylcholine (ACh) 1 µM for 8 min. After recording the ACh induced Ca^2+^ oscillatory activity, superfusion was switched back to HBSS-HEPES for 1 min. The biomaterials were inserted with transwell inserts (0.4 µm) using a special holder after 1 min and recorded for 30 min. Calcium imaging was performed on an inverted fluorescence microscope with an EM-CCD camera and FluoFrame imaging Software. Peak amplitude, time to maximum amplitude, number of Ca^2+^ transients of individual cells and area under the curve of the Ca^2+^ dynamics were calculated with Matlab 8.0 (The Mathworks Inc., Natick, MA, USA) software.

### qRT-PCR analysis

qRT-PCR analyses were done after 1 day. cDNA was produced and amplified using the Reverse Transcriptome kit (QuantiTect Reverse Transcription kit, Qiagen, Hilden, Germany). Target cDNA was amplified using specific primer pairs. qRT-PCR was performed using Sensimix SYBR No-ROX Kit (Bioline, London, UK) on Light cycler 480 System (Roche Life Science, Penzberg, Germany). Samples were normalized using qBasePlus (Biogazelle NV, Zwijnaarde, Belgium) against at least three of the following genes: *Rpl13a*, *Eif4b*, *B2m*, *Actb*, or *Gapdh* as described previously^55^. Details of specific primers used for gene expression analysis are provided in Table 1.

**Table 1:** List of primer sequences used in this study.

Target gene	Primer	
	Forward	Reverse
GAPDH	CTACCAGTGCAAAGAGCCCA	TGGTCATCAACCCTTCCACG
ACTB	CTTCGCGGGCGACGAT	CCACATAGGAATCCTTCTGACC
B2M	ACTTAGAGGTGGGGAGCAGA	GCCCTTTACACTGTGAGCC
EIF4b	GTGCGTTTACCACGTGAACC	CGTGCATCCTGGTCTGACTT
RPH3a	CTGGTCCGAGTTTTCTCCGC	TTCTTTATCATTTGATTGAAGGGGC
BGLAP	CTCACACTCCTCGCCCTAT	TCTCTTCACTACCTCGCTGC
TGF-β1	AGGGCTACCATGCCAACTTC	GACACAGAGATCCGCAGTCC
MMP1	CCCAGCGACTCTAGAAACACA	CTGCTTGACCCTCAGAGACC
BMP2	AGTCCTGATGAGCATGAGCC	CTCACCTATCTGTATACTGC

### Cytotoxicity assays

#### Live/dead staining

Live/dead staining was performed using calcein acetoxymethyl (AM) ester (Tebu-Bio, Boechout, Belgium) / propidium iodide (PI; Sigma-Aldrich, Overijse, Belgium). The samples were rinsed with 500 µL phosphate buffered saline (PBS). 500 µL PBS, 1 µL PI and 1µL calcein AM (Sigma-Aldrich, Overijse, Belgium) was added to each well and after 10 min incubation in a dark room, samples were evaluated with a fluorescence microscope (Type U-RFL-T, Olympus, Antwerpen, Belgium).

#### MTT assay

To quantify cell viability, the colorimetric 3-(4,5-dimethyl-2-thiazolyl)- 2,5-diphenyl-2H-tetrazolium bromide (methyl thiazolyl tetrazolium; MTT) assay was performed. The cell culture medium was replaced by 0.5 mg/ml MTT reagent and cells were incubated for 4 h at 37 °C. After removal of the MTT reagent, lysis buffer (1% Triton X-100 in isopropanol/0.04 N HCl) was added and incubated for 30 min at 37 °C on a gyratory shaker (70 rpm). The dissolved formazan solution was transferred into a 96-well plate and the optical densities (OD) were measured spectrophotometrically at 580 nm (Universal Microplate Reader EL 800, Biotek Instruments, Vermont, USA). Cell viability was calculated according to the following equation^56^:

$$ {\text{Cell}}\,{\text{viability}}\,(\% )  =  100 \times {\text{OD}}\,{\text{(mean}}\,{\text{of}}\,{\text{sample) / OD}}\,{\text{(mean}}\,{\text{of}}\,{\text{control)}}.$$

#### TUNEL assay

TUNEL assay kit (Roche, Basel, Switzerland) was used for staining TUNEL positive cells and DAPI nuclear stain was used for staining viable cells. The TUNEL positive and DAPI cells were visualized in fluorescence microscope (BD Pathway 435, BD Biosciences, New Jersey, USA) and images were analysed using Fiji Image J software^57^.

### Mineralization assay

The cells were seeded on plastic coverslips of 13 mm diameter (Nunc Thermanox, ThermoFisher, Massachusetts, USA) in an adhesive 24 well plate. Osteogenic medium was prepared by supplementing standard culture medium with 10 mM β-glycerophosphate (Sigma-Aldrich), 100 µM L-ascorbic acid 2-phosphate (Sigma-Aldrich) and 100 nM dexamethasone (Sigma-Aldrich). Sample extracts were prepared at a non-toxic dilution as determined by MTT assay. For both Alizarin Red and Von Kossa staining, either pure extract with standard culture medium or extract supplemented with osteogenic medium was added to the cells. At 7, 14, 21 and 28 days, Alizarin Red (Alizarin red dye 1 g in 40 ml ultra-pure water, VWR, Oud Heverlee, Belgium) and Von Kossa (silver nitrate 1 g in 20 ml ultra-pure water, VWR, Oud Heverlee, Belgium) staining were performed. After rinsing with PBS, the cells were fixed with neutral buffered formaldehyde. The reaction was accelerated by adding formaldehyde-sodium carbonate solution (1 g in 15 ml ultra-pure water, VWR, Oud Heverlee, Belgium). Unreduced silver ions were removed by Farmer’s solution (Sigma-Aldrich, Overijse, Belgium) containing 10% potassium ferrocyanide and 90% sodium thiosulfate. All images were captured with a microscope (Olympus BX51, Olympus, Tokyo, Japan) equipped with Xcellence software (Olympus, Tokyo, Japan).

### Statistical analysis

All data were subjected to statistical analysis by unpaired t-test with Welch's correction, analysis of variance (ANOVA) and individual comparisons were performed by Tukey post-hoc at a significance level of *p* < 0.05 using Statistical Package for Social Sciences (SPSS) v25.0 (IBM Corp., Armonk, NY, USA) and GraphPad Prism (version 6, GraphPad Software Inc., San Diego, CA, USA).

### Conference presentation

Parts of this study has been presented at the 6th Belgian Symposium on Tissue Engineering, 2018.

## Supplementary Information

Supplementary Figures.
