# Supplementary material for: The calcium dynamics of human dental pulp stem cells stimulated with tricalcium silicate-based cements determine their differentiation and mineralization outcome
Source: Sci Rep. 2021 Jan 12;11:645. doi: 10.1038/s41598-020-80096-5 (PMC7804324; doi:10.1038/s41598-020-80096-5)
Supplement: Supplementary file 1 — Supplementary Figures. [file 41598_2020_80096_MOESM1_ESM.docx]

***Supplementary figures***


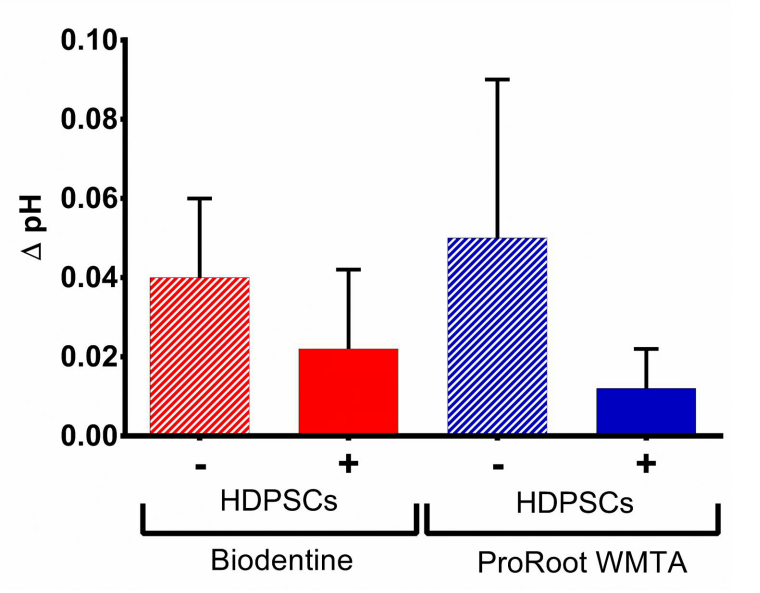


***Supplementary figure 1:*** *Rise in pH in α –MEM with and without hDPSCs after 30 minutes. No significant difference between the groups were observed.*


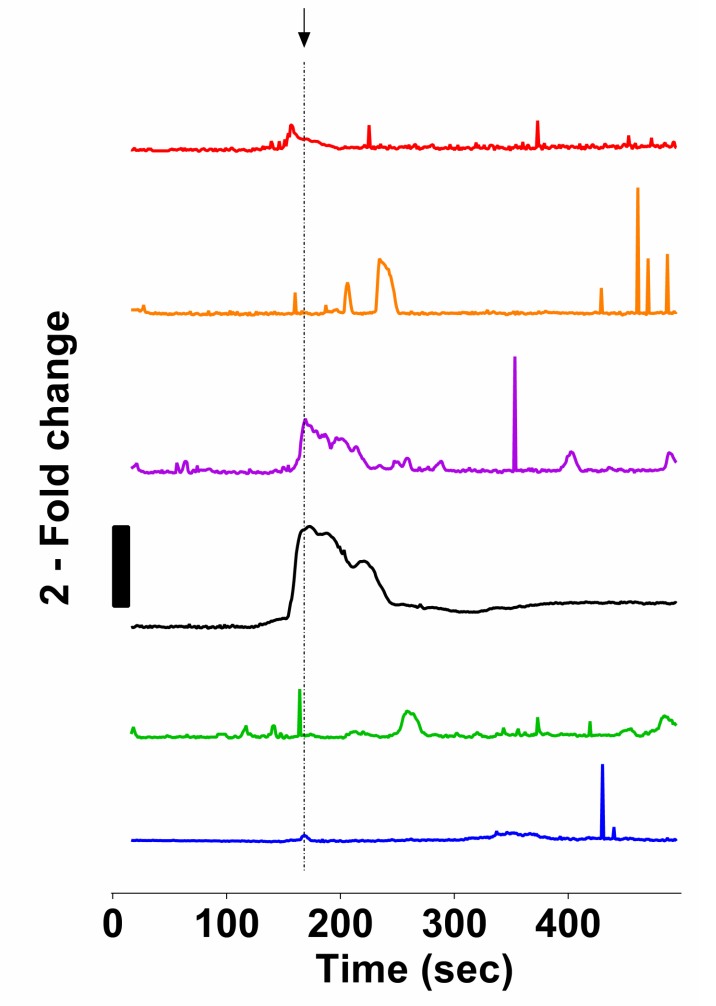


***Supplementary figure 2:*** *Representative graphs for intracellular Ca^2+^ dynamics induced by acetylcholine. The dotted line and arrow indicate the point of insertion of acetylcholine.*


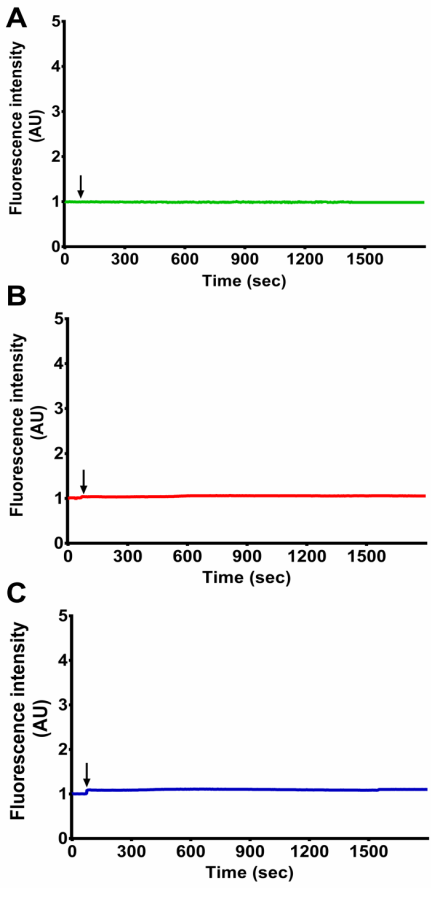


***Supplementary figure 3:*** *Representative intracellular Ca^2+^ traces recorded from cells pre-loaded with BAPTA-AM as a control condition. (A) Acetylcholine. (B) Biodentine. (C) ProRoot WMTA. Arrow indicates the point of insertion of acetylcholine/ biomaterial.*


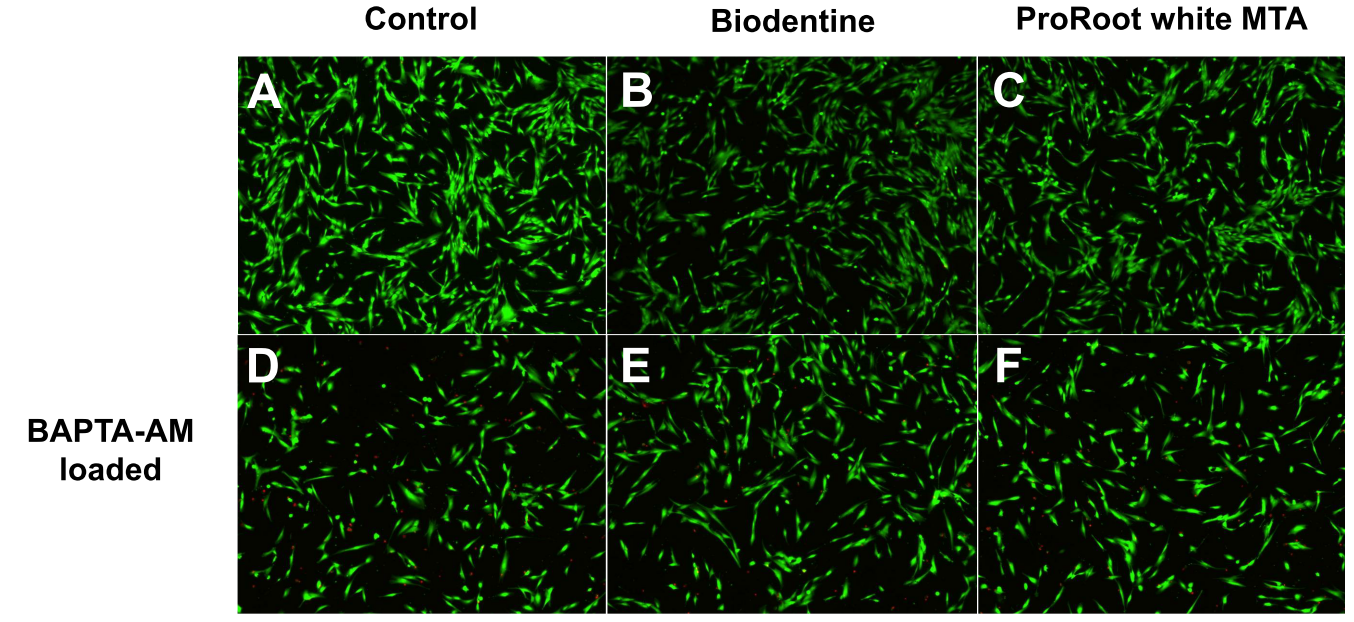


***Supplementary figure 4:*** *Figures A-F represent viability assay visualized by live/dead assay after 1 day. Green cells represent live cells and red cells represent dead* *cells.* *(A) Control (B) Biodentine (C) ProRoot WMTA (D) Control BAPTA-AM loaded (E) Biodentine BAPTA-AM loaded and (F) ProRoot WMTA BAPTA-AM loaded. No visual differences could be seen between the groups.*
